# FHOD1 is upregulated in glioma cells and attenuates ferroptosis of glioma cells by targeting HSPB1 signaling

**DOI:** 10.1111/cns.14264

**Published:** 2023-05-21

**Authors:** Fan Zhang, Lixiang Wu, Songshan Feng, Zijin Zhao, Kui Zhang, Abhimanyu Thakur, Zhijie Xu, Qiuju Liang, Yuanhong Liu, Wei Liu, Yuanliang Yan

**Affiliations:** ^1^ Department of Gynecology, Xiangya Hospital Central South University Changsha China; ^2^ Department of Physiology, School of Basic Medical Science Central South University Changsha China; ^3^ Department of Neurosurgery, Xiangya Hospital Central South University Changsha China; ^4^ State Key Laboratory of Silkworm Genome Biology, Medical Research Institute Southwest University Chongqing China; ^5^ Pritzker School of Molecular Engineering, Ben May Department for Cancer Research University of Chicago Chicago Illinois USA; ^6^ Department of Pathology, Xiangya Hospital Central South University Changsha China; ^7^ Department of Pharmacy, Xiangya Hospital Central South University Changsha China; ^8^ National Clinical Research Center for Geriatric Disorders, Xiangya Hospital Central South University Changsha China

**Keywords:** ferroptosis, FHOD1, glioma, HSPB1, prognosis

## Abstract

**Background:**

As a new type of regulatory cell death, ferroptosis has been proven to be involved in cancer pathogenesis and therapeutic response. However, the detailed roles of ferroptosis or ferroptosis‐associated genes in glioma remain to be clarified.

**Methods:**

Here, we performed the TMT/iTRAQ‐Based Quantitative Proteomic Approach to identify the differentially expressed proteins between glioma specimens and adjacent tissues. Kaplan–Meier survival was used to estimate the survival values. We also explored the regulatory roles of abnormally expressed formin homology 2 domain‐containing protein 1 (FHOD1) in glioma ferroptosis sensitivity.

**Results:**

In our study, FHOD1 was identified to be the most significantly upregulated protein in glioma tissues. Multiple glioma datasets revealed that the glioma patients with low FHOD1 expression displayed favorable survival time. Functional analysis proved that the knockdown of FHOD1 inhibited cell growth and improved the cellular sensitivity to ferroptosis in glioma cells T98G and U251. Mechanically, we found the up‐regulation and hypomethylation of HSPB1, a negative regulator of ferroptosis, in glioma tissues. FHOD1 knockdown could enhance the ferroptosis sensitivity of glioma cells via up‐regulating the methylated heat‐shock protein B (HSPB1). Overexpression of HSPB1 significantly reversed FHOD1 knockdown‐mediated ferroptosis.

**Conclusions:**

In summary, this study demonstrated that the FHOD1‐HSPB1 axis exerts marked regulatory effects on ferroptosis, and might affect the prognosis and therapeutic response in glioma.

## Graphical abstract

Our findings indicate that FHOD1 is upregulated in glioma cells. The glioma patients with low FHOD1 expression displayed favorable survival time. Knockout of FHOD1 could enhance the ferroptosis sensitivity of glioma cells.

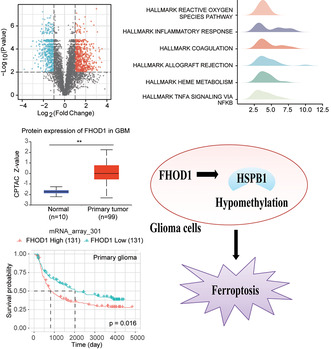

## 1 INTRODUCTION

As the most common and aggressive nervous system malignancy, gliomas represent more than 70% of primary brain tumors.^1^ Although advances in surgical excision and chemoradiotherapy, the therapeutic effect and prognosis of this disease remain unfavorable.^2^, ^3^, ^4^ Furthermore, cytopathological and genomic heterogeneity often contribute to the complicated pathogenesis of glioma.^5^ Thus, it is highly desired to clarify the novel molecular targets for personalized treatment and prognostic assessment in glioma patients. For example, strategies are currently being made to identify the underlying regulators for the induction of cell ferroptosis.^6^

The formin family members, including the formin homology 2 domain‐containing protein 1 (FHOD1), could act as the capping and bundling modulators facilitating to remodel the actin cytoskeleton.^7^ Recently, aberrated FHOD1 has been proven to be participate in several cancer‐associated processes. Overexpression of FHOD1 (1–339) domains caused a remarkable decrease in the centrosome separation in osteosarcoma cells U2OS.^8^ High levels of FHOD1 promoted cancer cell migration and invasion by inducing the ability of epithelial‐mesenchymal transition in squamous cell carcinoma.^9^ The actin‐regulating formin protein FHOD1 was frequently overexpressed in several breast cancer cells. Knockdown of FHOD1 significantly interfered with the invasion, migration, and proliferation of breast cancer cell MDA‐MB‐231.^10^ In addition, the findings from Ménard's group^11^ preliminarily indicated the potential roles of FHOD1 c‐terminal cleavage fragments during cell death. Thus, the identification of FHOD1 and its associated signaling networks underlying cell death would provide a suitable basis to sensitize cancer cells to cell death‐based therapeutic strategies.

As a new type of regulatory cell death, ferroptosis has been proven to be triggered by the following biological processes, such as depletion of glutathione, intracellular iron accumulation, lipid peroxidation, and production of cellular reactive oxygen species (ROS).^12^, ^13^, ^14^ Nowadays, emerging reports have pointed out the important roles of ferroptosis in the tumorigenesis and treatment of cancers, including glioma.^15^, ^16^ A risk signature containing seven ferroptosis‐related genes (FRGs) could be used to effectively predict the glioma patients' prognosis and immune status.^17^ The hypoxic condition could significantly suppress the sulfasalazine‐induced ferroptosis by upregulating the expression of solute carrier family 7 member 11 (SLC7A11) in glioma cells U87 and U251.^18^ Inhibition of solute carrier family 1 member 5 (SLC1A5) significantly confers the ferroptosis sensitivity by deactivating the GPX4‐dependent pathway and improving the therapeutic efficacy of anti‐PD‐1.^19^ Thus, clarifying the underlying regulatory mechanisms of ferroptosis could be a promising strategy for the clinical management of glioma patients.

In this study, we explored the functional roles of FHOD1 signaling in the regulation of ferroptosis in glioma cells. We found that FHOD1 was overexpressed in glioma cells. Silencing of FHOD1 remarkably promoted the ferroptosis sensitivity and growth inhibition in glioma cells through inhibiting heat‐shock protein B (HSPB1).

## 2 MATERIALS AND METHODS

### 2.1 Cell culture and reagents

Human glioma cells T98G and U251 were kindly acquired from Cancer Research Institute, Central South University, China.^20^ In brief, these cells were incubated in Dulbecco's modified eagle medium (DMEM, HyClone) with 10% fetal bovine serum (FBS, Bioind) and 1% penicillin–streptomycin (Gibco). These cells were cultured in a 37°C incubator with 5% CO_2_. Erastin was purchased from Sigma‐Aldrich. Ferrostatin‐1 (Fer‐1) was purchased from APExBIO. The working concentrations of erastin and Fer‐1 in glioma cells were 10 and 1 μM, respectively.

### 2.2 Lentiviruses

The FHOD1 shRNAs were purchased from Sigma. FHOD1 shRNA‐1: TCTACGAGAACGCCCTGAAAT and FHOD1 shRNA‐2: TGGCCCACAGTGACACTATTC. We co‐transfected TransIT‐X2 (MIRUS Bio) with shRNA vector, packaging plasmid psPAX2, and envelope plasmid pMD2.G into human embryonic kidney cells HEK293T for about 48 h. The lentivirus‐containing media was then collected after transfection. The viruses and polybrene (8 μg/mL) were mixed to infect the glioma cells for about 48 h. These infected cells were harvested for subsequent analysis.

### 2.3 Identification of differentially expressed proteins

Four pairs of fresh glioma specimens and adjacent tissues were collected from the Department of Neurosurgery, Xiangya Hospital, Central South University (Changsha, China). And we also obtained informed consent. This study has been approved by the Ethical Committee of Xiangya Hospital, Central South University. Total proteins were extracted in lysis buffer (Thermo Scientific) supplemented with the protease inhibitor cocktails (B14012, Bimake) for 15 min. Then, the equal protein solutions were subjected to TMT/iTRAQ‐Based Quantitative Proteomic Approach (PTMBio) to identify the differentially expressed proteins.

### 2.4 Quantitative reverse‐transcription polymerase chain reaction

The transcription levels of HSPB1 were evaluated by quantitative reverse‐transcription polymerase chain reaction (qRT‐PCR). The total RNA from FHOD1‐depleted glioma cells was extracted using the Trizol reagents (Cat#15596018, Invitrogen). After reverse‐transcribed with PrimeScript 1st strand cDNA synthesis kit (Cat#6210A, Takara), we used qRT‐PCR to evaluate the HSPB1 expression at transcriptional levels. Relative mRNA levels were calculated by the 2^−ΔΔCT^ method. The primers for PrimPol are 5′‐ACGGTCAAGACCAAGGATGG‐3′ and 5’‐AGCGTGTATTTCCGCGTGA‐3′. The primers for Actin are 5′‐CATGTACGTTGCTATCCAGGC‐3′ and 5′‐CTCCTTAATGTCACGC ACGAT‐3′.

### 2.5 Immunoblotting

For western blot, the glioma cells were lysed in lysis buffer (Thermo Scientific) supplemented with the protease inhibitor cocktails (B14012, Bimake) for 15 min. The supernatants were collected after centrifugation for 15 min at 12,000 rpm. After then, the 50 μg total proteins were loaded on SDS‐PAGE and transferred onto the PVDF membranes (Millipore). After blocking in 5% skimmed milk for about 1 h, the PVDF membranes were incubated with the indicated antibodies overnight at 4°C. The indicated primary antibodies were as follows: anti‐FHOD1 (ab206692, 1:1000, Abcam), anti‐HSPB1 (18284‐1‐AP, 1:1000, Proteintech), anti‐TRF1 (11899‐1‐AP, 1:1000, Proteintech) and anti‐β‐actin (Sc‐69,879, 1:5000, Santa Cruz). The protein levels were determined by the Immobilon Western Chemiluminescent HRP Substrates (Millipore).

### 2.6 Cell counting kit‐8 (CCK‐8) assay

About 2 × 10^3^ cells were seeded into 96‐well plates after transfection with FHOD1 shRNAs or Flag‐HSPB1 for about 24 h. After then, the transfected cells were treated with the indicated concentrations of erastin or Fer‐1 for 24 h at 37°C. After incubating with 10 μL Cell counting kit‐8 (CCK‐8) reagent (B34304, Bimake) for 1 h, the optical density values of glioma cells were identified at 450 nm using VICTOR™ X2 microplate reader (PerkinElmer).

### 2.7 Colony formation assay

Colony formation assay was conducted to measure the effects of the FHOD1‐HSPB1 axis on cell survival. In brief, after transfection with FHOD1 shRNAs or Flag‐HSPB1 for about 48 h, 1× 10^3^ cells were seeded in six‐well plates and sequentially incubated for 10–15 days at 37°C to facilitate the colony formation. After being fixed with 100% ethanol, the cell colonies were stained with 0.006% crystal violet solution and counted. The surviving colonies were defined as the colonies consisting of more than 50 cells.

### 2.8 Immunohistochemical staining

The glioma tissue microarrays were purchased from OUTDO BIOTECH (HBraG180Su02). After carefully checking, the number of available samples is 145. In addition, 50 formalin‐fixed, paraffin‐embedded (FFPE) specimens of glioma tissues were collected from the Department of Pathology, Xiangya Hospital, Central South University (Changsha, China). And this study has been approved by the Ethical Committee of Xiangya Hospital, Central South University. The immunohistochemistry (IHC) analysis was conducted as previously described.^21^, ^22^ In brief, the IHC Select® HRP/DAB kit (Millipore, # DAB150) was used to conduct the immunohistochemical staining of FHOD1 (1:150) and HSPB1 (1:150). The staining intensity was scored by two independent pathologists, and divided as 0 (negative), 1 (weak), 2 (moderate), and 3 (strong). The χ^2^ test was utilized to analyze the correlation between FHOD1 levels and patients' stages or grades.

### 2.9 Measurement of intracellular iron

Iron assay kit (Ab83366, Abcam) was used to detect the concentration of irons in glioma cells. Upon treatment with erastin and Fer‐1 for 24 h, glioma cells were collected and mixed with iron assay buffer rapidly. After removing the insoluble material at 14,000 × g for 15 min, the iron probes were added to the reaction mixture. At last, the absorbance of the stable‐colored complex was detected at 593 nm using VICTOR™ X2 microplate reader (PerkinElmer).

### 2.10 Measurement of cellular ROS

DCFDA/H2DCFDA Kit (ab113851, Abcam) was used to detect the cellular ROS levels. In the dark condition, 20 μM DCFDA Solution was added to stain the glioma cells for 30 min at 37°C. After then, the cellular fluorescence signal was immediately detected at 535 nm using flow cytometer.

### 2.11 Glioma xenograft models

All nude mice were maintained and manipulated based on the guidelines approved by the Ethical Committee of Xiangya Hospital, Central South University. We injected 5 × 10^6^ glioma cells (T98G‐shNC, T98G‐shFHOD1, or T98G‐shFHOD1 + HSPB1) into the healthy adult BALB/C nude mice (5 weeks), to construct the glioma xenograft models. The formula (length × width × height) × (π/6) was used to calculate the tumor volumes. After the tumor volumes reached approximately 100 mm^3^, we euthanized the mice and removed the tumor.

### 2.12 Statistical analysis

The Student's *t*‐test with SPSS15.0 software was used to conduct the statistical analysis between the two groups. A *p*‐value of less than 0.05 was considered significant. A *p*‐value of less than 0.01 was considered very significant. All data are presented as the mean ± SD.

## 3 RESULTS

### 3.1 FHOD1 expression was up‐regulated in glioma

**FIGURE 1:**
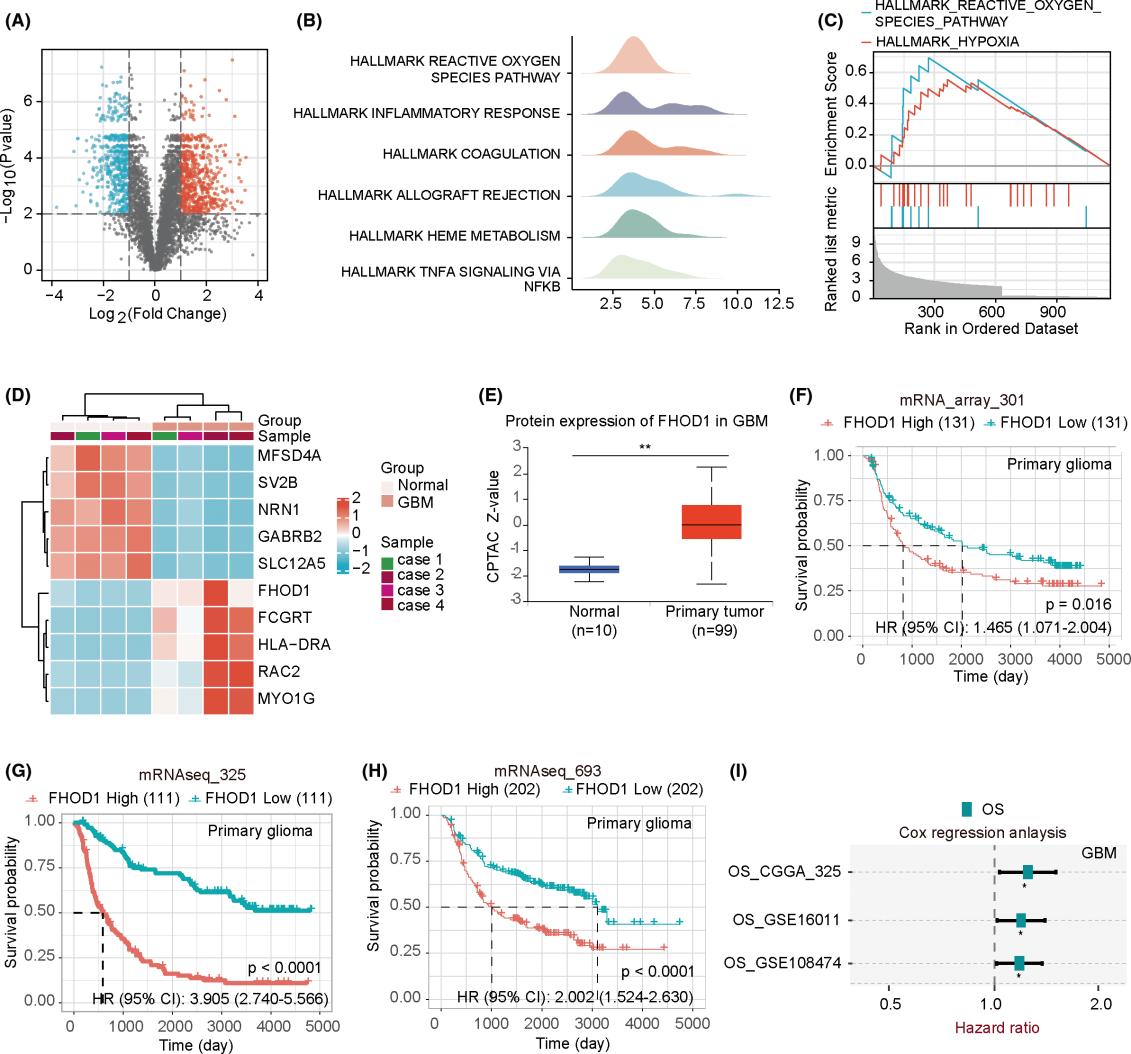
Identification of up‐regulated FHOD1 expression in glioma tissues. (A) Volcanic maps suggested the up‐regulated and down‐regulated molecules in glioma tissues. (B, C) GSEA analysis indicated the significant pathways regulated by the differentially expressed molecules. (D) Heat map of the top 10 altered molecules. (E) The CPTAC database confirmed up‐regulated FHOD1 in glioma tissues. (F–H) In three CGGA datasets, the glioma patients with low FHOD1 expression displayed favorable prognosis. (I) The glioma patients with high FHOD1 expression displayed high risk of recurrence.

We used the TMT/iTRAQ‐Based Quantitative Proteomic Approach to identify the differentially expressed proteins between glioma specimens and adjacent tissues. Using the screening criteria of fold change >2 and *p* value <0.05, we identified 1117 differentially expressed proteins (635 upregulated and 542 downregulated) in the glioma samples (Figure 1A and Table S1). Using GSEA analysis, we confirmed that the ROS pathway might be the significant pathway regulated by these differentially expressed proteins (Figure 1B,C). Heat map of the top 10 altered proteins indicated that FHOD1 was the most significantly upregulated protein (Figure 1D), suggesting FHOD1 is a promising predictive biomarker for glioma. Pancancer analysis revealed the up‐regulated FHOD1 in several cancers, including renal clear cell carcinoma, pancreatic adenocarcinoma, and glioma (Figure S1A). The Chinese Glioma Genome Atlas (CGGA)^23^ was used to confirm the positive association between FHOD1 levels and patients' grades in three datasets, mRNA_array_301, mRNAseq_325, and mRNAseq_693, (Figure S1B–D). Next, we utilized Clinical Proteomic Tumor Analysis Consortium (CPTAC) from the University of ALabama at Birmingham CANcer data analysis Portal (UALCAN)^24^ to demonstrate that FHOD1 was up‐regulated in glioma tissues (Figure 1E). CGGA database was further used to evaluate the effects of aberrantly expressed FHOD1 on the patients' prognosis. In three datasets from the CGGA database, mRNA_array_301, mRNAseq_325, and mRNAseq_693, the glioma patients with low FHOD1 expression displayed favorable overall survival (OS) (Figure 1F–H). Moreover, in CGGA_325, GSE16011^25^ and GSE108474,^26^ the patients with high FHOD1 expression all displayed high risk of recurrence (Figure 1I). Taken together, these results indicated that the high expression of FHOD1 was associated with poor outcomes in glioma patients.

### 3.2 Knockdown of FHOD1 inhibited the growth of glioma cells

**FIGURE 2:**
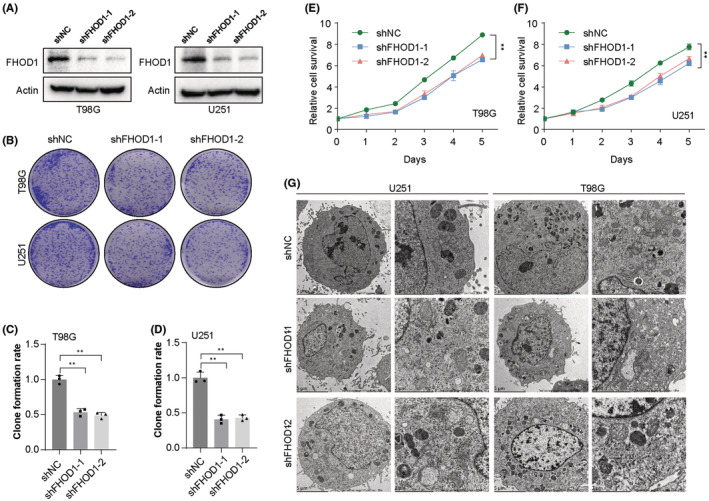
The inhibitory effect of FHOD1 knockdown on the growth of glioma cells. (A) Western blot confirmed the down‐regulation of FHOD1 expression in Ctrl or FHOD1 shRNAs stably‐expressed T98G and U251 cells. (B) Colony formation assay indicated the inhibitory effect of FHOD1 depletion on growth of glioma cells. (C, D) Quantification of cell survival determined by colony formation assay. (E, F) CCK‐8 assay indicated the inhibitory effect of FHOD1 depletion on proliferation of glioma cells. (G) The mitochondrial morphological changes revealed by electron microscope. Error bars represented the mean ± SD from three independent experiments. **p < 0.01.

Two glioma cells T98G and U251 were utilized to evaluate the roles of FHOD1 on cell growth. We first used the short hairpin RNAs (shRNAs)‐mediated knockdown strategy to downregulate FHOD1 expression in glioma cells T98G and U251 (Figure 2A). Colony formation assay indicated the inhibitory effect of FHOD1 depletion on the growth of glioma cells T98G and U251 (Figure 2B–D). The change in cell proliferation was measured by CCK‐8 cell viability assay. The results of Figure 2E,F showed that FHOD1 knockdown significantly inhibits the cell proliferation rate. Given the cellular metabolic organelle, mitochondria could display the important biological functions for cell proliferation,^27^ we next used transmission electron microscopy to evaluate the change in mitochondrial morphology. Accordingly, we found knockdown of FHOD1 perturbed the mitochondrial structure characterized by increased mitochondrial membrane density and reduced mitochondrial size (Figure 2G). Thus, these findings supported the growth‐promoting action of FHOD1 in glioma cells.

### 3.3 Knockdown of FHOD1 improved the cellular sensitivity to ferroptosis

**FIGURE 3:**
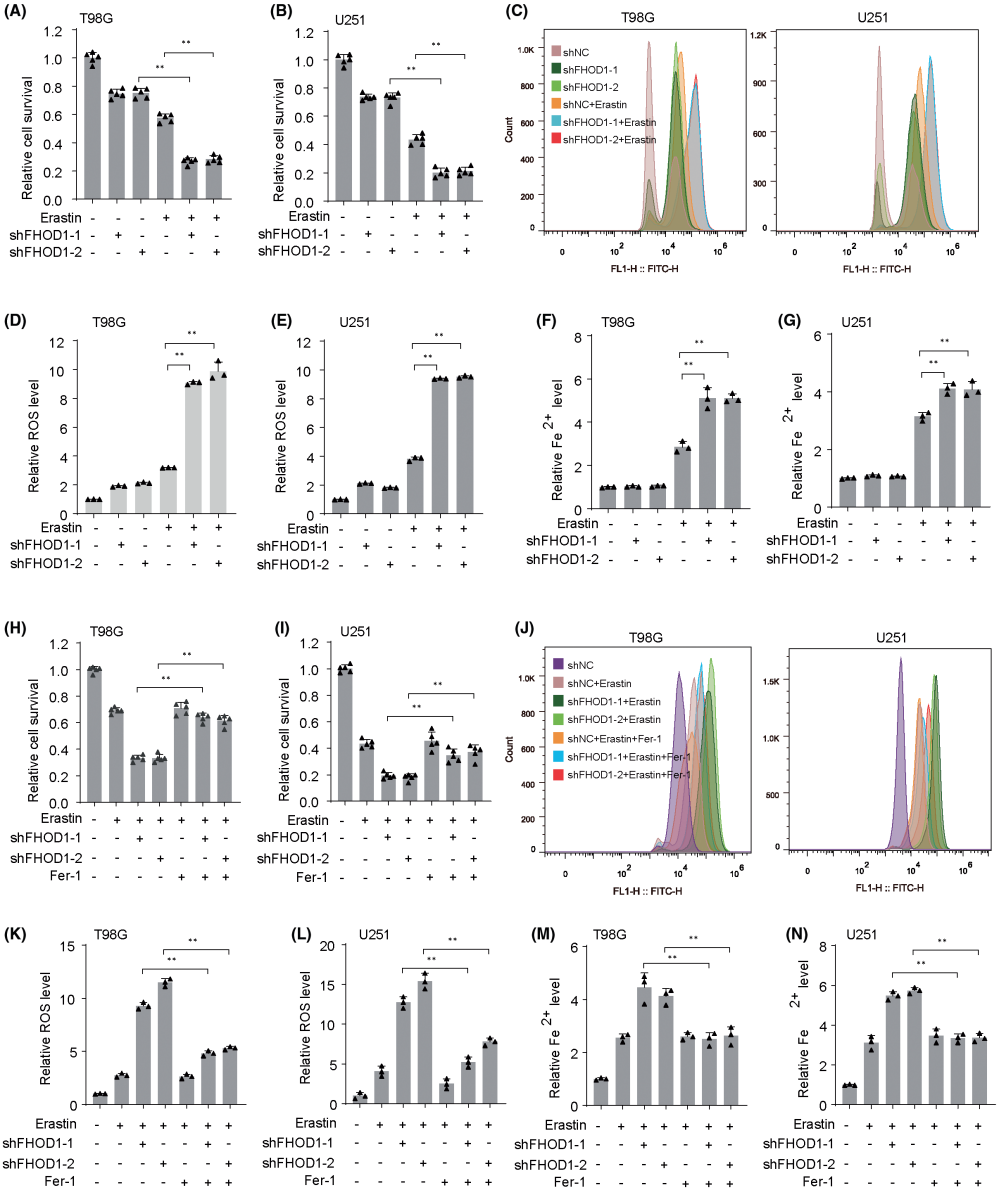
Knockdown of FHOD1 improved the ferroptosis in glioma cells. (A, B) CCK‐8 assay indicated the effects of FHOD1 knockdown on cytotoxic activity of erastin in glioma cells T98G and U251. (C) Flow cytometer indicated the cellular ROS fluorescence signal after FHOD1 knockdown. (D, E) Quantification of cellular ROS levels from (C). (F, G) The intracellular Fe2+ levels in FHOD1‐deficient T98G and U251 cells. (H, I) Ferroptosis inhibitor Fer‐1 antagonized the cytotoxic activity of erastin in FHOD1 knockdown T98G and U251 cells (J) Flow cytometer indicated the cellular ROS fluorescence signal after FHOD1 knockdown and Fer‐1 treatment. (K, L) Quantification of cellular ROS levels from (J). (M, N) The intracellular Fe2+ levels in FHOD1‐deficient T98G and U251 cells treated with Fer‐1. Error bars represented the mean ± SD from three independent experiments. **p < 0.01.

Emerging reports have sustained that activating ferroptosis could potentially inhibit the tumor growth.^28^, ^29^ Moreover, ferroptosis has been primarily characterized by cellular ROS production and iron concentration.^30^ Then, we would like to explore whether aberrant FHOD1 mediates ferroptosis of glioma cells. We first used the ferroptosis inducer erastin^31^ to treat the FHOD1‐depleted glioma cells T98G and U251. The CCK‐8 assay indicated that FHOD1 knockdown significantly increased erastin‐induced inhibition of proliferation in glioma cells T98G and U251 (Figure 3A,B). Moreover, administration of erastin significantly enhanced the accumulation of intracellular ROS and Fe2+ levels in FHOD1‐deficient T98G and U251 cells (Figure 3C–G). Conversely, treatment with ferrostatin‐1 (Fer‐1), a ferroptosis inhibitor,^32^ antagonized the growth‐inhibitory effect induced by erastin in FHOD1 knockdown T98G and U251 cells (Figure 3H,I). The up‐regulated trend of intracellular ROS and Fe2+ concentrations in FHOD1‐silenced cells were also markedly suppressed (Figure 3J–N). These findings suggested that FHOD1 promoted the ferroptosis resistance of glioma cells.

### 3.4 FHOD1 up‐regulated the ferroptosis‐associated HSPB1

**FIGURE 4:**
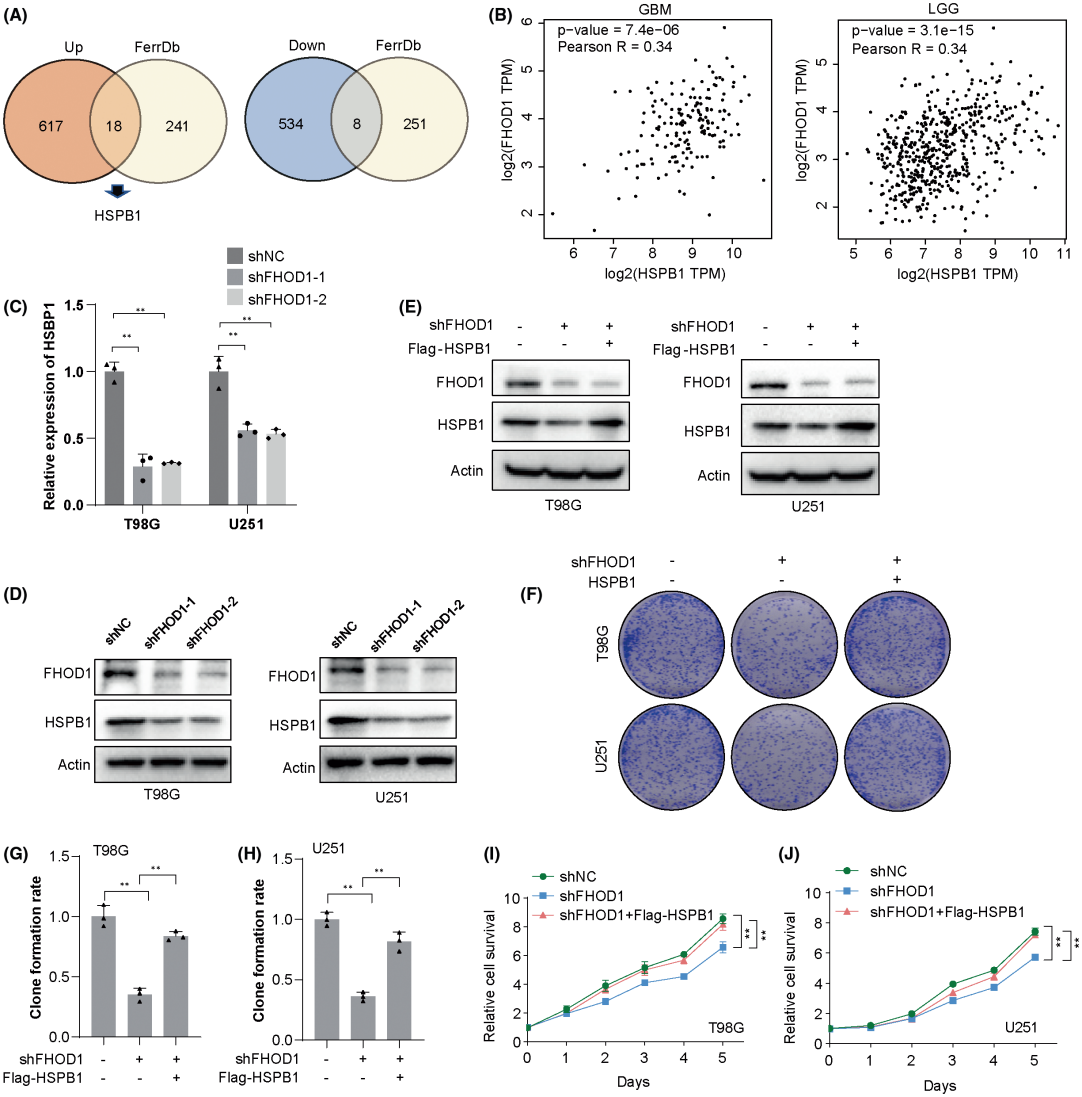
FHOD1 knockdown inhibited the ferroptosis‐associated HSPB1. (A) Venn diagrams identified the significantly upregulated HSPB1. (B) The correlation between FHOD1 expression and HSPB1 expression in GBM and LGG tissues. (C, D) Western blot and qRT‐PCR confirmed the down‐regulation of HSPB1 expression in FHOD1‐depleted T98G and U251 cells. (E) After FHOD1 knockdown and Flag‐HSPB1 overexpression, the total protein was extracted and analyzed by western blot with the indicated antibodies. (F) FHOD1‐depleted T98G and U251 cells reconstituted with Flag‐HSPB1 were used to detect the cell growth rates. (G, H) Quantification of cell growth determined by colony formation assay from (F). (I, J) FHOD1‐depleted T98G and U251 cells reconstituted with Flag‐HSPB1 were used to detect the cell proliferation rates. Error bars represented the mean ± SD from three independent experiments. **p < 0.01.

We utilized the Venn diagrams to overlap the 1117 differentially expressed proteins (Table S1) and ferroptosis‐related molecules (Table S2), and identified the significantly upregulated HSPB1, a negative regulator of ferroptosis^33^ (Figure 4A). Then, we explored whether FHOD1 regulated HSPB1‐dependent cell ferroptosis. Pearson correlation analysis indicated the positive correlation between FHOD1 expression and HSPB1 expression in both glioblastoma multiforme (GBM) and lower‐grade glioma (LGG) tissues (Figure 4B). Moreover, the expression levels of HSPB1 were found significantly reduced in FHOD1‐depleted glioma cells T98G and U251 (Figure 4C,D). In addition, HSPB1 was overexpressed in FHOD1‐deficient T98G and U251 glioma cells (Figure 4E). Colony formation and CCK‐8 experiments both suggested that ectopic expression of HSPB1 obviously blocked the growth‐inhibitory effect of FHOD1 knockdown in glioma cells (Figure 4F–J). These data collectively revealed that HSPB1 could be served as the downstream factor of FHOD1 in glioma.

### 3.5 The ferroptosis resistance‐induced by FHOD1 was dependent on HSPB1

**FIGURE 5:**
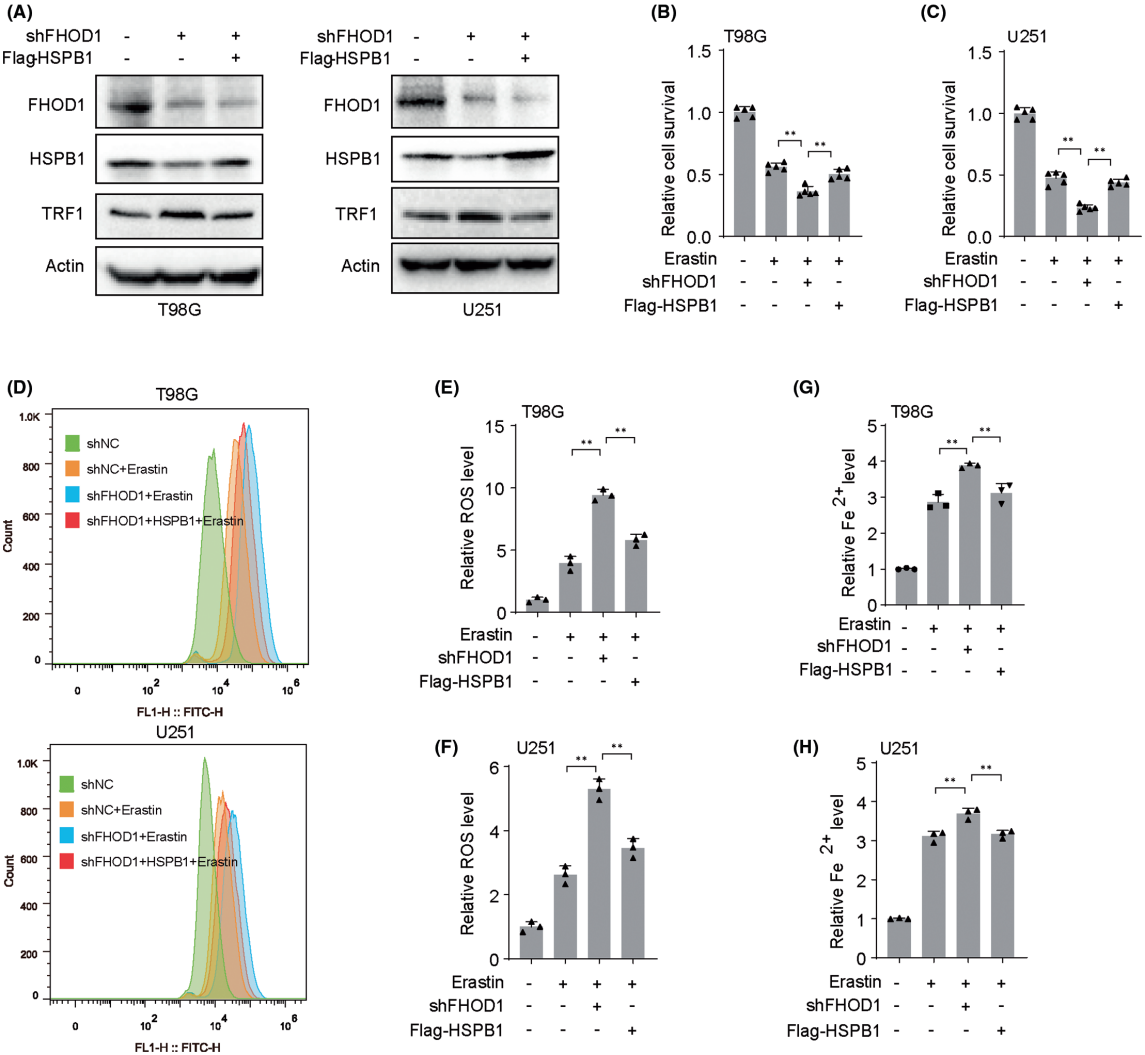
The ferroptosis resistance‐induced by FHOD1 was dependent on HSPB1. (A) After FHOD1 knockdown and Flag‐HSPB1 overexpression, the total protein was extracted and analyzed by western blot with the indicated antibodies. (B, C) CCK‐8 assay indicated the cytotoxic activity of erastin in T98G and U251 cells with FHOD1 knockdown and Flag‐HSPB1 overexpression. (D) Flow cytometer indicated the cellular ROS fluorescence signal after FHOD1 knockdown and Flag‐HSPB1 overexpression. (E, F) Quantification of cellular ROS levels from (D). (G, H) The intracellular Fe2+ levels in T98G and U251 cells with FHOD1 knockdown and Flag‐HSPB1 overexpression. Error bars represented the mean ± SD from three independent experiments. **p < 0.01.

Next, we would like to explore the effects of the FHOD1‐HSPB1 signaling axis in the regulation of ferroptosis. As shown in Figure 5A, in T98G and U251 glioma cells, HSPB1 overexpression suppressed the FHOD1 knockdown‐mediated upregulation of TRF1, a ferroptosis‐positive regulator.^34^ Moreover, overexpression of HSPB1 in FHOD1‐depleted glioma cells significantly reduced the inhibition of cell growth (Figure 5B,C). Simultaneously, the improved concentrations of intracellular ROS and Fe2+ induced by FHOD1 knockdown were markedly reversed upon HSPB1 overexpression (Figure 5D–H). Collectively, these findings suggested that FHOD1 protected glioma cells against ferroptosis via down‐regulating HSPB1 expression.

### 3.6 HSPB1 was hypermethylated induced by FHOD1 knockdown

**FIGURE 6:**
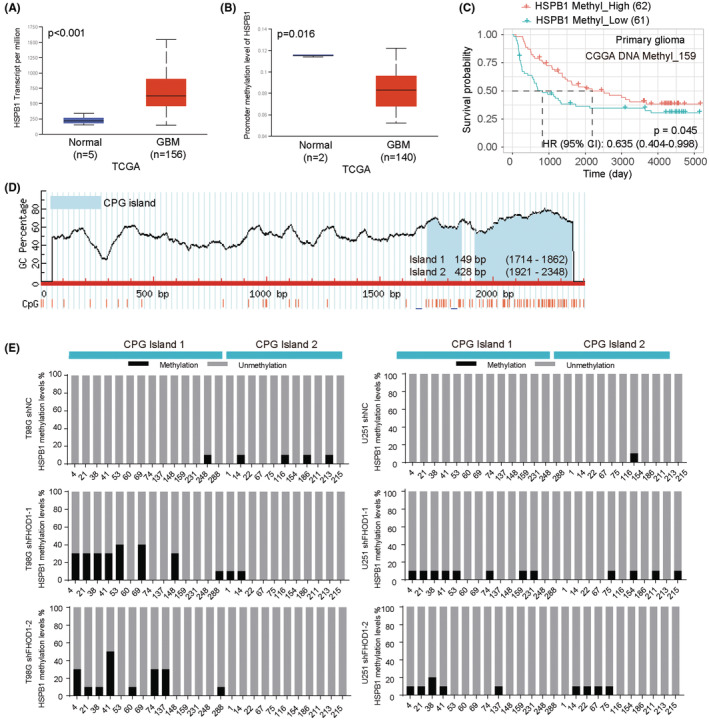
HSPB1 is hypomethylated in glioma cells. (A) UALCAN database indicated the up‐regulated HSPB1 mRNA levels in glioma tissues. (B) UALCAN database indicated the hypomethylated HSPB1 in glioma tissues. (C) The patients with hypermethylated HSPB1 displayed favorable prognosis. (D) Two CpG islands in HSPB1 gene promotor were identified by MethPrimer. (E) Bisulfite sequencing PCR was used to confirmed the methylated islands of HSPB1 promotor after FHOD1 knockdown.

Increasing reports have demonstrated that DNA methylation, an epigenetic mechanism, plays a promising role in the regulation of gene expression, and involves in the cancer pathogenesis and therapeutic response.^35^ We would like to explore whether the aberrant DNA methylation affect the expression of HSPB1. First, the CPTAC from the UALCAN database was used to demonstrate that HSPB1 expression was up‐regulated in glioma tissues (Figure S2A). In three datasets from the CGGA database, mRNA_array_301, mRNAseq_325, and mRNAseq_693, the glioma patients with high HSPB1 expression displayed unfavorable survival time (Figure S2B–D). After then, the UALCAN database was used to confirm the up‐regulation and hypomethylation of HSPB1 in glioma tissues (Figure 6A,B). The patients with hypermethylated HSPB1 displayed favorable survival time (Figure 6C). The CGGA database was used to show the negative association between HSPB1 methylation values and patients' grades (Figure S2E). In addition, by using MethPrimer, we identified two CpG islands located at the HSPB1 gene promotor, island 1 (1714–1862), and island 2 (1921–2348) (Figure 6D). Bisulfite sequencing PCR was used to confirm the improved methylated island 1 (1714–1862) after FHOD1 knockdown (Figure 6E). These results indicated FHOD1 knockdown could down‐regulated HSPB1 expression by promoting HSPB1 promoter hypermethylation.

### 3.7 The clinical significance of FHOD1–HSPB1 axis

**FIGURE 7:**
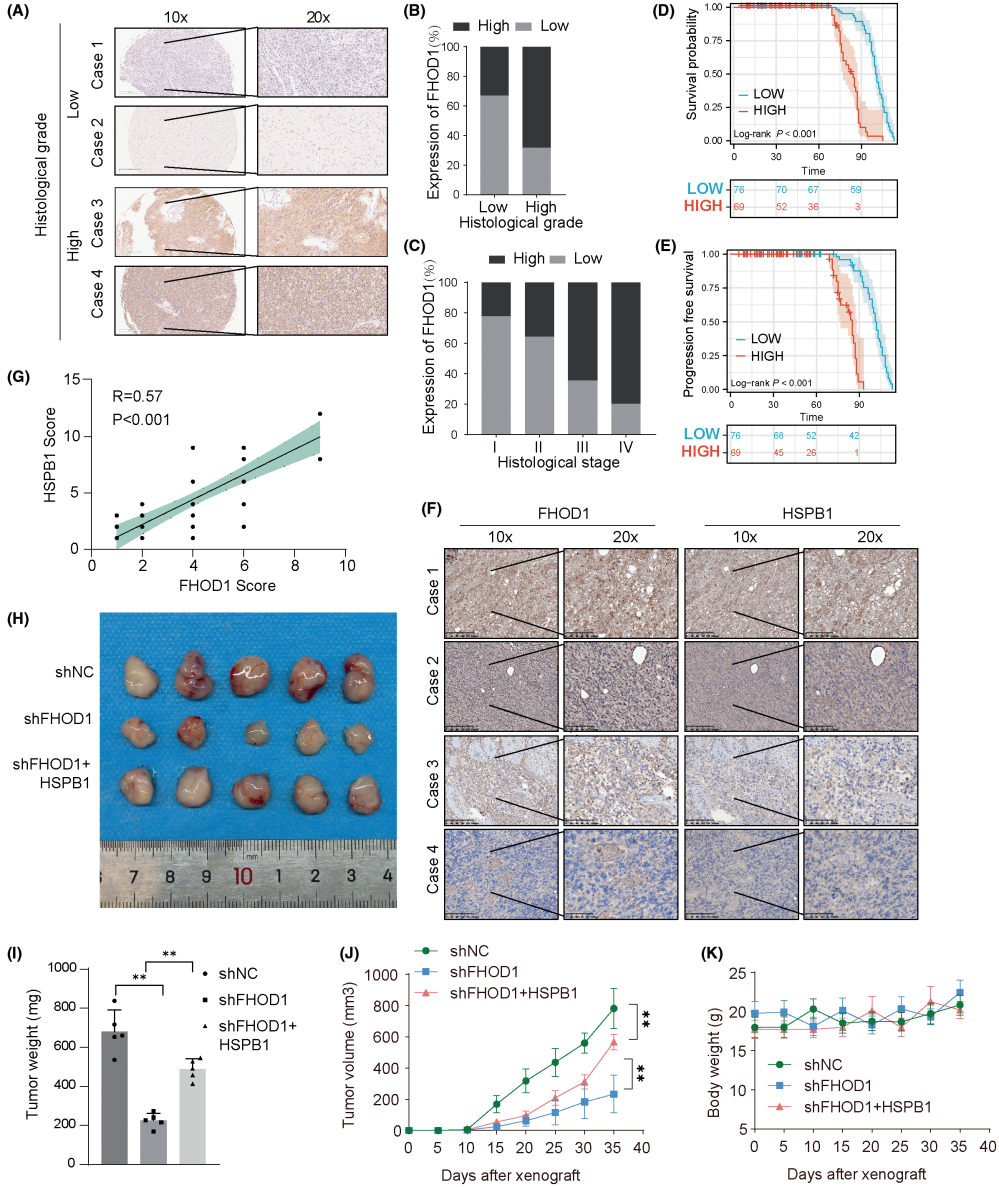
The clinical significance of FHOD1 in glioma. (A) The representative immunohistochemical staining of FHOD1 on glioma tissue microarray (*n* = 145). Scale bars are indicated. (B, C) Protein levels of FHOD1 was quantified in glioma specimens with different grades and stages. (D) The glioma patients with low FHOD1 expression displayed favorable OS. (E) The glioma patients with low FHOD1 expression displayed favorable PFS. (F) The representative immunohistochemical staining of FHOD1 on Xiangya glioma cohorts (*n* = 50). Scale bars are indicated. (G) Correlation analysis of FHOD1 and HSPB1 in glioma samples. Statistical analyses were performed with the χ^2^ test. The Pearson *r* indicates correlation coefficient. (H) The tumor tissues were harvested at the end of the experiment in each group. (I, J) The tumor weight and tumor volume of each group in glioma xenograft models. The asterisks (**) indicate the following: FHOD1 knockdown vs. control (*p* < 0.01) and FHOD1 knockdown + HSPB1 overexpression vs. FHOD1 knockdown (*p* < 0.01). (K) The body weight curves of each group in glioma xenograft models.

We performed immunohistochemical staining of FHOD1 on a glioma tissue microarray. The representative images of lowly‐stained and highly‐stained FHOD1 are shown in Figure 7A. Most tumor tissues from advanced grade and stage patients showed high levels of FHOD1. We observed a significant positive correlation between FHOD1 levels and the patients' grades (Figure 7B) and stages (Figure 7C). Moreover, the glioma patients with high FHOD1 expression displayed unfavorable OS (Figure 7D) and PFS (Figure 7E), which were similar to Figure 1F–H. These data suggested the oncogenic roles of FHOD1 in glioma patients.

Next, we would like to confirm the biological roles of the FHOD1‐HSPB1 axis in glioma. We first explored the expression correlation between FHOD1 and HSPB1 proteins in glioma cohorts from Xiangya Hospital, Central South University. The representative images of lowly‐stained and highly‐stained FHOD1/HSPB1 are shown in Figure 7F. And we found a positive correlation between FHOD1 and HSPB1 expression levels (*p* < 0.001, Pearson *r* = 0.57; Figure 7G). Second, we performed the glioma xenograft models in nude mice to explore the anti‐glioma effects of FHOD1‐HSPB1 signaling. As shown in Figure 7H–J, depletion of FHOD1 inhibited the tumor volume and weight, indicating a tumor‐promoting role of FHOD1 in glioma. However, the cytotoxic effects of FHOD1 knockdown could be significantly reversed by Flag‐HSPB1 overexpression. In addition, treatment with FHOD1 knockdown and HSPB1 overexpression could not cause the changes of body weight in xenograft models (Figure 7K). Taken together, our results suggested that the FHOD1‐HSPB1 axis may be a potential target for glioma research and treatment.

## 4 DISCUSSION

In this study, we first studied the biological significance of FHOD1 in the regulation of ferroptosis in glioma cells. These findings confirmed that FHOD1 was markedly increased in glioma tissues and cell lines. Knockout of FHOD1 significantly enhanced the ferroptosis sensitivity by weakening HSPB1 expression.

Ferroptosis, a new type of programmed cell death, has been proven to be induced by the overloading of cellular iron and ROS.^36^ During the past decades of research, the regulatory factors of ferroptosis have been proposed to participate in the gliomagenesis and anti‐tumor responses.^37^ In glioma cells U251 and U87, overexpression of NEDD4L significantly reinforced the cytotoxic effects induced by the natural compound paeoniflorin (PF), accompanied by inhibition of cell viability and induction of ferroptosis.^38^ Upon fear of stress, METTL3 upregulation could enhance the FSP1 stability, resulting in glioma progression and ferroptosis resistance.^39^ Acting as a tumor‐suppressive circRNA, circLRFN5 overexpression could significantly improve ferroptosis sensitivity, consequently impairing the cell viabilities and tumorigenesis of glioma stem cells.^40^ Accordingly, we found that FHOD1 knockdown significantly increased elastin‐induced ferroptosis in glioma cells T98G and U251. The results suggested that FHOD1‐associated signaling pathways might play an essential role in the regulation of ferroptosis resistance.

HSPB1, a recently identified ferroptosis‐associated gene,^41^ has been proved to be involved in human diseases, including cancers. Overexpression of HSPB1 exhibited a neuroprotective effect in rats with hypoxic–ischemic brain damage through attenuating cell ferroptosis.^42^ Overexpression of circST6GALNAC6 could improve the ferroptosis sensitivity in bladder cancer cells by blocking the HSPB1‐P38 signaling axis.^33^ A novel risk signature containing ferroptosis‐associated HSPB1 has been established and could be used to predict the survival time and radiosensitivity in glioma patients.^43^ Here, we demonstrated the up‐regulated HSPB1 expression in glioma tissues. The glioma patients with high HSPB1 expression displayed unfavorable survival time. In addition, HSPB1 overexpression could markedly restrained the accumulation of intracellular ROS and Fe2+ induced by FHOD1 knockdown. Moreover, the pro‐ferroptotic effects of FHOD1 knockdown in glioma cells could be effectively impaired by HSPB1 overexpression in vitro and in vivo. All these findings suggested the depletion of FHOD1 could increase the ferroptosis sensitivity of glioma cells via inhibiting HSPB1 signaling.

In summary, our research mainly indicated FHOD1 as a promising negative regulator for ferroptosis in glioma. Clarifying the understanding of molecular mechanisms and biological functions of FHOD1 in glioma biology would be of great significance to improve the prognosis and therapeutic response.

## AUTHOR CONTRIBUTIONS

FZ and YY: conception and design. LW, SF, ZZ, KZ, AT, ZX, QL, YL, and WL: data curation. YY: writing the manuscript and revision of the manuscript. All authors contributed to the article and approved the submitted version.

## FUNDING INFORMATION

This study is supported by grants from the National Natural Science Foundation of China (82272659), the Science and Technology Innovation Program of Hunan Province (2021RC3029).

## CONFLICT OF INTEREST STATEMENT

The authors declare that there are no conflicts of interest.

## Supporting information


Figure S1.

Figure S2.

Table S1:

Table S2:

## Data Availability

The data generated from this study are available upon request from the corresponding authors.
