# Supplementary figures and images for: FHOD1 is upregulated in glioma cells and attenuates ferroptosis of glioma cells by targeting HSPB1 signaling
Source: CNS Neurosci Ther. 2023 May 21;29(11):3351–63. doi: 10.1111/cns.14264 (PMC10580363; doi:10.1111/cns.14264)

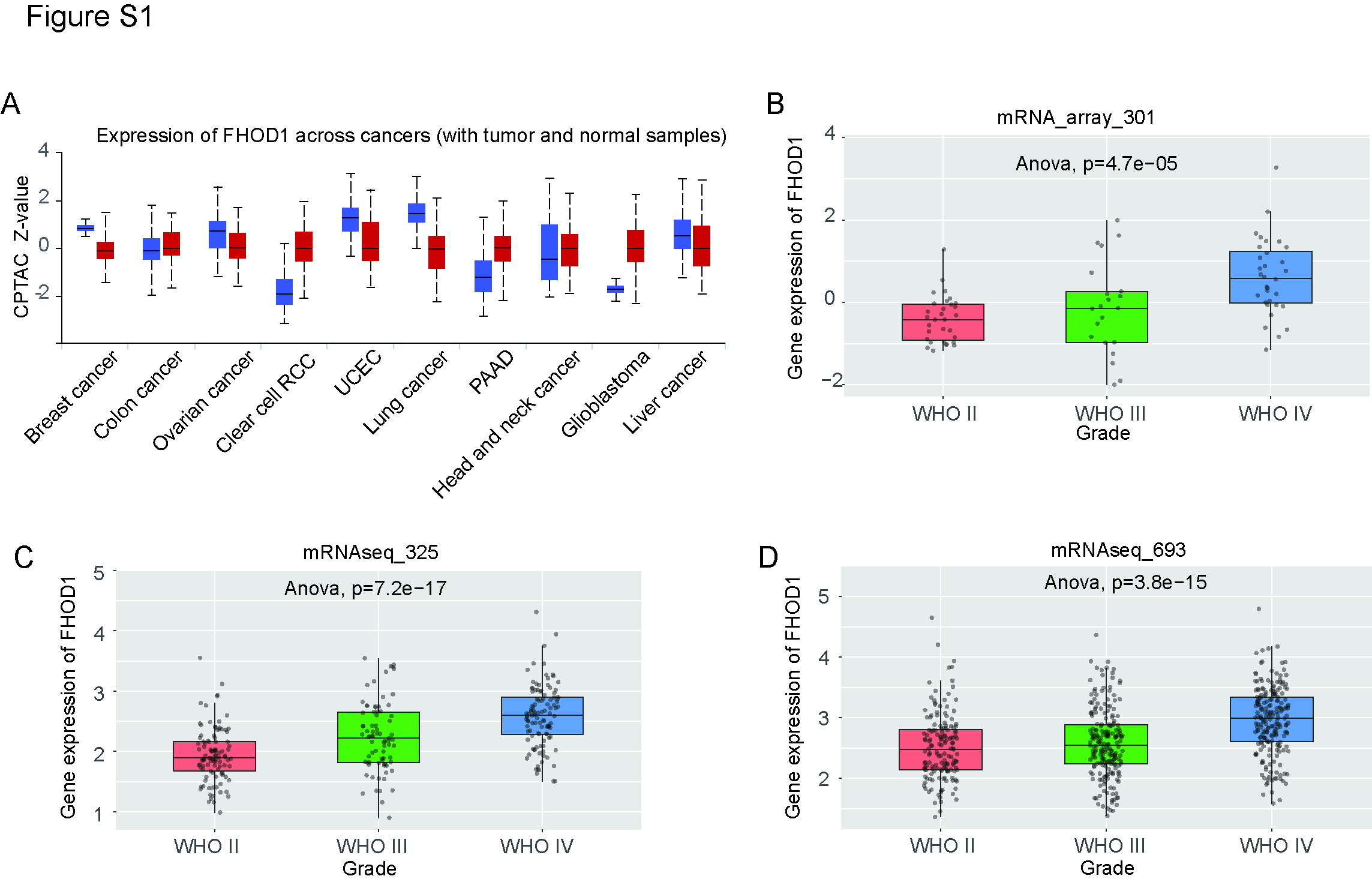

Supplement: Supplementary file 1 — Figure S1. [file CNS-29-3351-s003.tif]

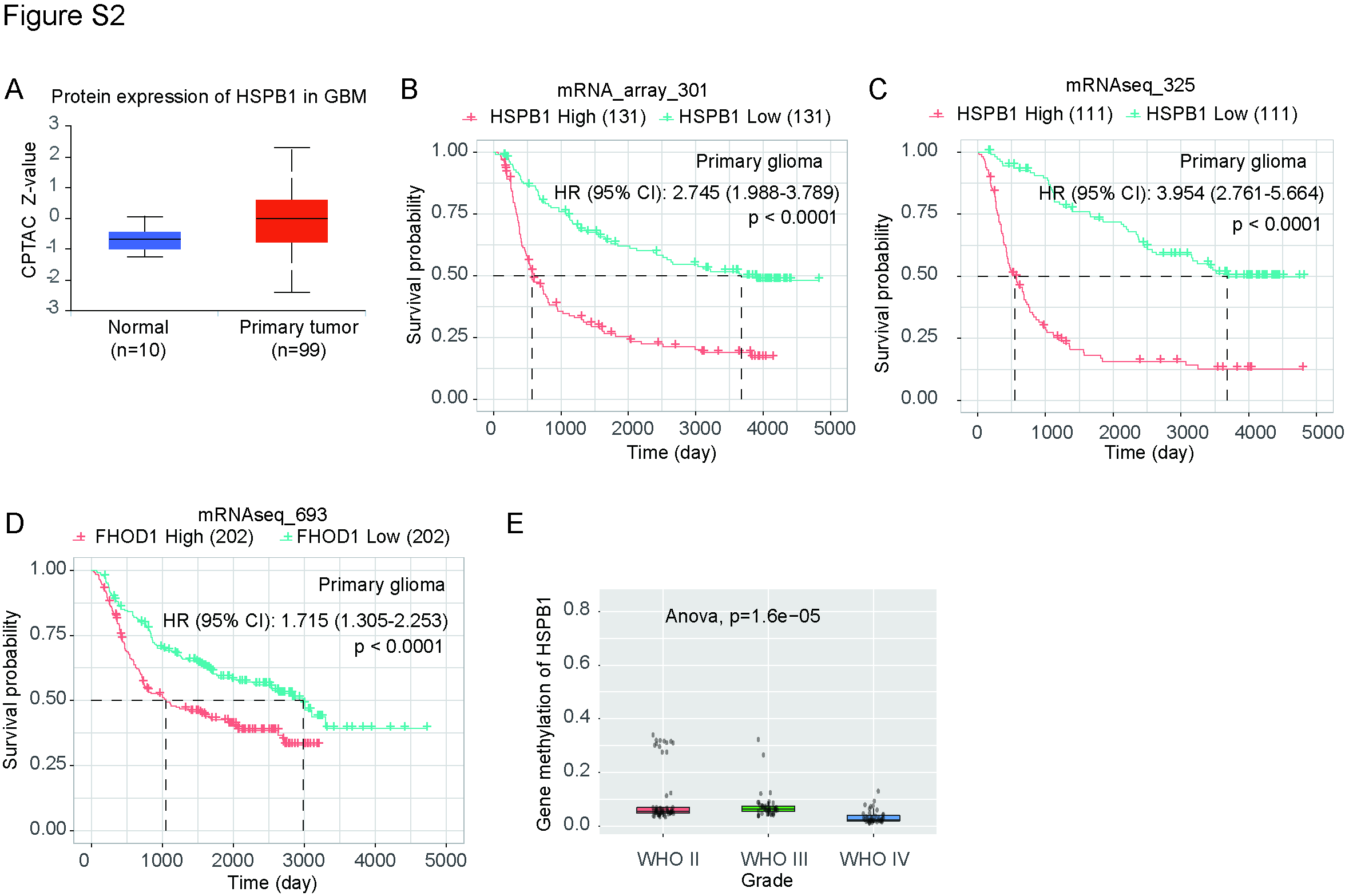

Supplement: Supplementary file 2 — Figure S2. [file CNS-29-3351-s004.tif]
